# Referral experiences of healthcare consumers: results from a cross-sectional study in urban slums in southeast Nigeria

**DOI:** 10.3389/fpubh.2025.1561158

**Published:** 2025-05-27

**Authors:** Chinelo Obi, Ifeyinwa Arize, Chukwudi Nwokolo, Benard Okechi, Bassey Ebenso, Obinna Onwujekwe

**Affiliations:** ^1^Health Policy Research Group, University of Nigeria, Enugu, Nigeria; ^2^Department of Health Administration and Management, University of Nigeria, Enugu, Nigeria; ^3^Centre for Entrepreneurship and Development Research, University of Nigeria, Enugu, Nigeria; ^4^Department of Psychology, University of Nigeria, Enugu, Nigeria; ^5^Leeds Institute of Health Sciences, University of Leeds, Leeds, United Kingdom

**Keywords:** healthcare system, healthcare consumers, healthcare providers, referral, urban slums, informal health providers

## Abstract

**Introduction:**

The inadequate referral system in Nigeria is partly due to the proliferation of informal healthcare providers (IHPs) and constraints within formal providers in urban slums. With limited data on patient referral experiences across these providers, this paper explores referral experiences in urban slums in southeast Nigeria.

**Methods:**

This study involved 1,025 people in eight slums in Enugu and Anambra states, using multi-stage purposive sampling. Data on referral experiences were collected through a pre-tested questionnaire and analyzed using descriptive statistics and logistic regression. Ethical approval was obtained, and informed consent was secured.

**Results:**

It found that only 7.4% of patients received referrals from their primary healthcare sources, mostly from patent medicine vendors, private clinics, and primary healthcare (PHC) centres to private clinics and laboratories. Verbal referrals were the dominant modality, although the types of referrals varied significantly between facilities that initiated referrals and between states.

**Conclusion:**

This paper highlights the need for innovative solutions to integrate informal healthcare providers into the formal system, improving referrals and enhancing health services in urban slums.

## Introduction

The referral pattern within the Nigerian health system is weak and lacks proper continuity across the three healthcare levels and is thus ineffective (1). The three levels of care in the Nigerian health system are the primary, secondary and tertiary levels. The major challenge across the three levels of healthcare is low levels of referrals from lower to higher levels of care (2–5). Theoretically, an integrated referral system is supposed to connect these levels of care with the primary healthcare level as the first contact for patients. If a case is too complex, it should be referred to secondary care, and if needed, further referred to tertiary hospitals for specialized treatment (1, 6).

The referral pattern fails, especially in urban slums and rural areas where formal healthcare providers—such as public primary healthcare (PHC) centers, public and private hospitals, private clinics, and pharmacies—are scarce. In these areas, informal healthcare providers (IHPs) are the main source of care. The referral system often fails in urban slums and rural areas due to a lack of formal healthcare providers, which includes the public PHC centres, public and private hospitals, leaving IHPs as the main source of care. IHPs include patent medicine vendors (PMVs), traditional birth attendants (TBAs), herbalists, bonesetters and drug sellers. They operate outside the formal healthcare system, further weakening referrals between primary healthcare centres and hospitals (6).

The almost non-existent formal referrals from the IHPs to the formal healthcare providers leads to limited access to timely and appropriate healthcare, especially for those in underserved areas like urban slums and rural communities (7). Hence, improving proper referrals from IHPs to formal providers is crucial, especially in urban slums where IHPs that mostly provide poor quality and inappropriate treatment are prevalent (8–10). Thus, closely integrating formal and informal care using the referral system is essential for creating an efficient, effective, and sustainable referral system (11).

A poor referral system may result from inadequate knowledge of the healthcare providers, patients’ non-compliance with referrals, patients’ preferences, financial barriers, and insufficient support (11). Additional challenges include poor road networks, lack of awareness about health facilities, poor coordination among providers, and inadequate training (11).

Improving referrals from IHPs to the formal system in urban slums by understanding and solving health providers and consumers constraining factors is key to better health for slum dwellers and the poor (12, 13). This aligns with the people-centered health systems (PCHS) approach, which enhances referral, promotes stronger links between formal and informal care, and ensures continuity of care at different levels and sites (12, 14). The model of patient-centred care (PCC) emphasizes that healthcare should respect and respond to individual patient preferences, needs, and values, ensuring that patient values guide all clinical decisions (15).

There is a shortage of knowledge about the drivers of poor referrals from the perspective of health consumers, particularly in low-resource and marginalized urban settings (4). Most existing research on referral systems in low- and middle-income countries (LMICs) tends to prioritize the perspectives of healthcare providers or system-level inefficiencies, with limited attention paid to the voices of patients navigating these systems (16, 17). Yet, patients’ lived experiences are critical for understanding gaps in referral uptake, including sociocultural, economic, and systemic barriers that hinder compliance (18). Exploring the experiences of healthcare consumers with the referral process can help in a better understanding of their perception and willingness to comply with referral recommendations. Incorporating health consumers’ perspectives into improving the referral systems aligns with having a PCHS promoting more effective, and personalized care (19). Also, engaging with patients and addressing their concerns can build this trust, leading more individuals to use formal referral pathways (20). Poor referral practices can lead to distrust in the formal healthcare system among residents of urban slums in Nigeria, reducing their likelihood of seeking formal care (20).

The aim of this research was to provide evidence on the often-overlooked referral experiences of healthcare consumers in urban slums in southeast Nigeria, examining their interactions with both formal and informal healthcare providers. This information is required to examine how referrals are undertaken in the health system, especially amongst IHPs in urban slums.

## Methods

### Study area

This cross-sectional study was conducted in Enugu and Anambra states, southeast Nigeria, with populations of 4.2million and 3.3million, respectively, at an annual average growth rate of 2.2% (21, 22). A total of eight (8) urban slums, four urban slums in each state were purposely selected considering the relative size of the slum and the availability of a functional primary health centre (PHC) in the slum.

Table 1 shows the formal and informal healthcare providers that provide care in urban slums.

**Table 1:** Types of formal and informal health providers in urban slums.

Formal health providers	Informal health providers
Government-owned primary healthcare centres	Drug hawkers
Private clinics	Spiritual healing homes
Private laboratories	Traditional birth attendants
	Herbalists

### Study population

The study population was predominantly female primary caregivers, mainly wives, mothers, and grandmothers. The sampling method for the survey was a multi-stage purposive sampling. The first stage involved the purposive selection of six (6) local government areas to ensure inclusiveness and representative selection. The second stage involved purposive selection of 8 slums, with 4 coming from each of the 2 states selected. Slum selection was based on the relative size of the slum (with the largest selected), the ones with functional primary health centre and perceived acceptability of the slums especially in terms of safety and security. The last stage involved the selection of households to be enumerated. This was done with selection of households with women of reproductive age (15 to 49 years) and households with at least a child of under 5 years of age. At the third stage, households were eligible if they had at least one of two criteria: a woman of reproductive age or a child under five. The selection of the household was facilitated with the help of community gatekeepers in each selected slum. The sample size of 1,025 households was determined using the guidelines outlined in the Demographic and Health Survey (DHS) listing manual (23).

### Data collection

Data was collected using a pre-tested interviewer-administered questionnaire. The questionnaire was used to collect data on the participants’ sociodemographic characteristics, and their referral knowledge and experiences while receiving healthcare in urban slums. Data on referral practices were collected from consumers who had visited healthcare facilities in the past three months. We inquired about the facilities that made and received referrals, the process of referral initiation, and how the referring healthcare provider communicated with the receiving facility. The questionnaire is available from the authors on request.

### Data analysis

Consumer characteristics, number of referrals, source of referral, referred facilities, type of referral, and responsibility of appointment were analyzed using frequencies and percentages. A cross-tabulation was undertaken between the referral facilities and the referred facilities, the referral facilities and the type of referral as well as between the referral facilities and the responsibility of appointment. A binary logistic regression was used to explore the socio-economic and health-seeking determinants of consumers getting referrals to higher levels of healthcare providers from their initial point of seeking care.

### Binary logistic model

The model was used to identify the socio-economic and health-seeking factors influencing households’ referrals to higher-level healthcare providers. The dependent variable was binary: coded as 1 for referred households and 0 for those not referred. Key independent variables included the state of residence (1 for Anambra, 2 for Enugu), household size (number of members), sex of the interviewed household member (1 for male, 2 for female), employment status (0 for not employed, 1 for employed), marital status (0 for not married, 1 for married), the type of healthcare facility typically visited (1 for formal, 2 for informal, 3 for both), and asset ownership (an index based on ownership of items like TVs, radios, and vehicles).

### Ethical considerations

Ethical clearance for the study was obtained from the University of Nigeria Teaching Hospital Health Research Ethics Committee (NHREC/05/01/2008B-FWA00002458-IRB00002323). Informed written consent was obtained from all participants, having informed them of study objectives, benefits and risks of participating in the study, and their rights to voluntary participation and confidentiality of data.

### Findings

A total of 1,025 questionnaires were collected and analyzed with 95.9% of respondents being females. Table 2 shows that 47.9% of the participants were female heads of household, followed closely by wives at 44.8%. The mean number of people in each household was 5 people and the average age of the respondents was 32.2 years. 99.2% of the respondents had attained a formal education (Table 2).

**Table 2:** Demographic characteristics of respondents.

Consumers characteristics	Total *n* (%) *N* = 1,025
Status in the household	
Female head of house	491(47.9)
Wife	459 (44.8)
Male head of house	36(3.5)
Grandmother	2(0.2)
Representative of household	37 (3.6)
Number of people in the household: mean (SD)	5.28(1.83)
Age: mean (SD)	32.17 (7.51)
Gender	
Female	983(95.9)
Male	42 (4.1)
Marital status	
Divorced/separated	21 (2.0)
Married	918 (89.6)
Never married	49 (4.8)
Widowed	37 (3.6)
Had formal education	1,017 (99.2)
Highest education level	
Junior secondary	123(12.0)
Primary	110 (10.7)
Senior secondary	618 (60.3)
Some primary	7 (0.7)
Teachers Training College	23 (2.2)
University	121 (11.8)
Other	15 (1.5)
Occupation (employed or unemployed)	
Employed	126 (12.3)
Unemployed	899 (87.7)

Table 3 shows that 7.4% of the respondents utilized referral services in the last three months. Majority (31.6%) of the referrals were from patent medicine vendors. A total of 39.5 and 38.2% of the referrals were from IHPs to private clinics and private laboratories, respectively. Approximately 60% of referrals were communicated verbally, while 36.8% were issued a referral slip to the designated facility. It was also found that 92% of the respondents booked an appointment with the facility that they were referred to.

**Table 3:** Utilization of referral services.

Description	Total *n* (%) *N* =
Consumers of healthcare services that were referred to another facility	76 (7.4%)
Facilities that initiated referral	
Drug hawkers/vendor	1 (1.3)
Government-owned General hospital (Secondary health facility)	4 (5.3)
Government-owned Primary health centre	14 (18.4)
Government-owned Tertiary health facility	2 (2.6)
Maternity home	2 (2.6)
Patent medicine vendor (PMV/Patent Medicine dealer/Chemist)	24 (31.6)
Pharmacy shop	10 (13.2)
Private clinic/hospital	17(22.4)
Private Laboratory	1 (1.3)
Traditional healing home	1 (1.3)
Where consumers of healthcare services were referred to	
Government owned General hospital (Secondary health facility)	5 (6.6)
Government owned Primary health centre	3 (4.0)
Government owned Tertiary health facility	5 (6.6)
Patent medicine vendor (PMV/Patent Medicine dealer/Chemist)	3 (4.0)
Pharmacy shop	1 (1.0)
Private clinic/hospital	30 (39.5)
Private Laboratory	29 (38.2)
Type of referral	
Verbal	46 (60.5)
Written	28 (36.8)
Phone number	2 (2.6)
Responsibility of appointment	
Appointment was booked	6 (7.9)
Consumers took responsibility for the referral	70 (92.1)

Table 4 shows that there was no significant relationship between the facilities that initiated referral and the referred facilities. However, the odds ratio shows that informal facilities are more than two times more likely to refer to formal facilities than to informal facilities.

**Table 4:** Referral facility and where they referred patients.

	Referred	Facilities				
Referral facilities	Informal	Formal	Total	Chi-square value	Odds ratio	Confidence interval
Informal	2 5.3%	36 94.7%	38 100%	0.347 (0.56)	2.056 (0.566)	0.1755893–24.06358
Formal	1 2.6%	37 97.4%	38 100%			
Total	3 3.9%	73 96.1%	76 100%			

Table 5 shows that more informal providers practiced verbal referrals while more formal providers practiced written referrals. There was an association between the facilities that initiated referrals and the type of referral that was given.

**Table 5:** Referral facility and type of referral.

	Type of	Referral			
Referral facility	Verbal	Written	Phone number	Total	Chi-square value
Informal	30 78.9%	8 21.1%	0	38 100%	11.4 (0.003^*^)
Formal	16 42.1%	20 52.6%	2 5.3%	38 100%	
Total	46 60.5%	28 36.8%	2 2.6%	76 100%	

* = means that the Chi-square test result is statistically significant (with *p* = 0.003), and the asterisk is used to visually flag this significance.

Table 6 shows no association between the referral facilities and taking responsibility for booking appointments for referred patients.

**Table 6:** Referral facility and responsibility of booking referral appointment.

	Responsibility of appointment			
Referral facilities	Appointment was booked	Consumers took responsibility for their appointment	Total	Chi-square value
Informal	1 2.6%	37 97.4%	38 100%	2.9 (0.09)
Formal	5 13.2%	33 86.8%	38 100%	
Total	6 7.9%	70 92.1%	76 100%	

Table 7 highlights the socio-economic and health-seeking determinants of households who sought care and were referred to a higher point of care. The results show that households in Enugu slums are 209% more likely to receive a referral than those in Anambra State (*p* < 0.01). Also, higher-income households are significantly (*p* < 0.01) more likely to be referred by 17%.

**Table 7:** Socio-economic and health seeking determinants of households getting referrals to higher levels of healthcare providers from their initial point of seeking care.

Variables		Odds ratio	Z stat.	P > |t|	95% conf. Interval
State (Enugu)		3.91	4.47	0.00*	2.15–7.12
Household size		0.91	−1.44	0.15	0.79–1.04
Sex of household representative (female)		1.18	0.29	0.77	0.40–3.46
Age		1.05	0.34	0.73	0.97–1.04
Employment status (employed)		1.72	1.10	0.27	0.65–4.55
Marital status (currently married)		0.97	−0.07	0.95	0.40–2.36
Facility type	Only informal facility	0.51	−0.93	0.35	0.13–2.08
	Combination of formal and informal facility	1.63	0.95	0.34	0.59–4.51
Asset ownership of household		1.83	4.30	0.00*	1.39–2.42
Constant		0.02	−4.00	0.00*	0.002–0.12
Prob > chi2		0.00			
Pseudo R-Square		0.10			
Number of observation		1,025			

// * = significant at 5%.

## Discussion

The focus on urban slums provides a unique perspective often overlooked in broader studies. Unlike previous studies that primarily focused on rural areas or general urban populations, this research sheds light on the unique issues faced by slum dwellers (24). It provides detailed data on the demographics, health-seeking behaviors, and referral experiences of residents in these underserved areas. This focus is crucial, as urban slums often experience more severe healthcare access issues, which intensify the negative effects of poor referral systems.

The findings of this study confirm the presence of inadequate referral practices and negative referral experiences in urban slums in southeast Nigeria. These results are consistent with previous studies in Nigeria, which have highlighted deficiencies in the referral system and low utilization, particularly from the perspective of healthcare providers (2, 3). Additionally, limited awareness among healthcare consumers about referral processes contributes to the problem (4).

The finding that consumers often bypassed PHC centers, which are intended as the first point of care, to self-refer to higher levels of healthcare in Nigeria (25), can be attributed to the poor conditions of PHC centers in urban slums (25). The substandard services from these PHC centres are attributed to incessant strikes among health workers, unavailability of PHC workers, dilapidated facility buildings, ill-equipped laboratories, lack of or inappropriate healthcare financing and remuneration for the health workers in the facilities (26). Furthermore, bureaucracy in the health system and the lackadaisical attitude of the healthcare workers lead to long waiting time in the health system, further discouraging patients from accessing services at the PHC centres (27, 28).

It was interesting to find PMVs as the most common source of referrals to the formal health system. This is intuitive because PMVs are often the first point of care in Nigeria (2, 6) and they frequently refer patients to PHC centers when they are not able to provide the requested services or when complications arise (29).

The finding that most referrals from the IHPs in the slums were to private clinics and private laboratories indicates a preference for private facilities over public ones. This trend may stem from the perception that private providers are better equipped and more readily available than public facilities (30). Prior studies support this notion, suggesting that the increased reliance on private providers is often due to the lack of essential equipment and medications in public facilities (31).

The findings reveal that both formal and informal healthcare providers in urban slums predominantly provide referrals using verbal communication, with healthcare consumers responsible for accessing and scheduling appointments. This reinforces the existence of an unstructured and weak referral system in the country (6).

Additionally, the study indicates that wealthier households are referred more frequently, likely due to their greater willingness and ability to seek healthcare early (32), even at the slightest symptoms. This reflects a trend where access to referral services is more about financial capability than actual need, reinforcing disparities in healthcare access (33, 34).

Although this study offers new insights into the specific challenges faced by healthcare consumers in urban slums, it has some limitations. It primarily gathered opinions from healthcare consumers, mainly female respondents, and did not include healthcare providers. Consequently, factors influencing providers’ choices of referral facilities were not examined. Additionally, the study was confined to urban slums in two states in southeast Nigeria, which may limit the generalizability of its findings.

In conclusion, poor referral practices, especially from IHPs to formal healthcare providers in urban slums hinder the continuum of care, resulting in a weak health system. Furthermore, since informal health providers are often the first point of contact in these communities, equipping them with training on effective referral practices is crucial. Strengthening formal linkages between informal and formal health systems could also enhance service delivery. Further studies should include equal representation of male and female service users and combine quantitative and qualitative methods to provide deeper insights into the challenges and opportunities for improving referrals, especially from IHPs to the formal health system.

## Data Availability

The raw data supporting the conclusions of this article will be made available by the authors, without undue reservation.
